# Acupuncture for Acne Vulgaris: A Systematic Review and Meta-Analysis

**DOI:** 10.1155/2018/4806734

**Published:** 2018-03-12

**Authors:** Suzi S. Y. Mansu, Haiying Liang, Shefton Parker, Meaghan E. Coyle, Kaiyi Wang, Anthony L. Zhang, Xinfeng Guo, Chuanjian Lu, Charlie C. L. Xue

**Affiliations:** ^1^China-Australia International Research Centre for Chinese Medicine, School of Health and Biomedical Sciences, RMIT University, P.O. Box 71, Bundoora, VIC 3083, Australia; ^2^Guangdong Provincial Hospital of Chinese Medicine, Guangdong Provincial Academy of Chinese Medical Sciences and The Second Clinical College, Guangzhou University of Chinese Medicine, Guangzhou, China

## Abstract

**Purpose:**

To conduct a systematic review and meta-analysis to determine the current best available evidence of the efficacy and safety of acupuncture and related therapies for acne vulgaris.

**Methods:**

Eleven English and Chinese databases were searched to identify randomized controlled trials (RCTs) of acne vulgaris compared to pharmacotherapies, no treatment, and sham or placebo acupuncture. Methodological quality was assessed using Cochrane Collaboration's risk of bias tool. Meta-analysis was conducted using RevMan software.

**Results:**

Twelve RCTs were included in the qualitative review and 10 RCTs were included in meta-analysis. Methodological quality of trials was generally low. The chance of achieving ≥30% change in lesion count in the acupuncture group was no different to the pharmacotherapy group (RR: 1.07 [95% CI 0.98, 1.17]; *I*^2^ = 8%) and ≥50% change in lesion count in the acupuncture group was not statistically different to the pharmacotherapy group (RR: 1.07 [95% CI 0.98, 1.17]; *I*^2^ = 50%).

**Conclusions:**

While caution should be exercised due to quality of the included studies, acupuncture and auricular acupressure were not statistically different to guideline recommended treatments but were with fewer side effects and may be a treatment option. Future trials should address the methodological weaknesses and meet standard reporting requirements stipulated in STRICTA.

## 1. Introduction

Acne vulgaris (acne) is a chronic and self-limiting condition that begins in adolescence and can last over 10 years [1]. Acne is characterized by inflamed and noninflamed comedones, oily skin, and cysts [2]. The mechanisms for the initial development of comedones are not fully understood [3]. Four factors have been identified which contribute to acne lesions and are the main targets of treatment. These factors include follicular keratinization, sebum production,* Propionibacterium acnes (P. acnes)*, and inflammatory mediator release [4]. Acne lesions may involve cellular inflammation causing hyperkeratinization of follicular ducts [5].* P. acnes* can induce keratinocytes to produce cytokines which rupture ducts, causing comedones [3]. Genetics [6] and androgen imbalances [7] can influence sebaceous gland lipid synthesis. Exacerbation can result from single or multiple factors such as* P. acnes*, menstruation, occupation, personal sweating, diet, or stress [2, 8].

Treatment of acne includes topical benzoyl peroxide and topical retinoids or antibiotics for mild to moderate acne and oral antibiotics combined with either topical benzoyl peroxide or topical or oral retinoids for severe acne [4]. Acupuncture is an umbrella term for traditional Chinese medicine techniques that stimulate acupuncture points. Techniques include acupuncture (insertion of fine needles at specific loci typically for a period of 20 to 30 minutes), auricular acupuncture (insertion of needles in specific loci of the auricle), auricular acupressure (placement of blunt instruments such as small metallic ball bearings at specific loci of the auricle), electroacupuncture (mild electric stimulation of acupuncture needles) [9], and moxibustion (burning of* Artemisia argyi *Levl. et Vant or* Artemisia vulgaris* leaf in a processed form) [10]. Several studies have suggested a potential role of acupuncture techniques in acne. Auricular acupressure and surrounding needle (where two to four needles are inserted superficially around the acne lesion) have been shown to reduce serum excretion rate (SER) and testosterone [11]. When acupuncture was combined with benzoyl peroxide, SER in women was reduced compared to benzoyl peroxide alone [12]. In animal studies, auricular acupuncture, auricular electroacupuncture, body acupuncture, and electro-acupuncture have been shown to decrease inflammation [13–16]. Auricular acupuncture may reduce acne inflammation through peripheral muscarinic receptors [13] and innate and adaptive immune responses [14, 17, 18], thereby possibly reducing acne inflammation.

Several reviews have examined the potential benefits of acupuncture techniques in clinical studies. A Cochrane review on complementary therapies for acne [19] evaluated efficacy of herbal medicine, acupuncture, cupping therapy, dietary modifications, purified bee venom, and tea tree oil. The review found there was a lack of evidence to support the use of herbal medicine and acupuncture. Two systematic reviews of acupuncture for acne have been published, one in English [20] and one in Chinese [21]. Cao et al. [20] included trials which used acupuncture, cupping, and other herbal medicines. While the number of “cured” cases increased when acupuncture was combined with cupping, or oral or topical herbal medicines, no benefit was found when acupuncture was compared with pharmacotherapy. The reviewers described the methodological quality of the papers as poor. Li et al. [21] included trials of manual acupuncture compared to routine conventional medicine (isotretinoin and antibiotics) or multiple Chinese medicine therapies. The authors were unable to provide conclusions due to the poor quality of the included trials.

These reviews included herbal medicines and techniques not commonly used outside of China. Acupuncture is commonly used in clinical practice for skin conditions, yet a gap exists in the evaluation of efficacy and safety of acupuncture for acne vulgaris. This review will analyze acupuncture compared to pharmacotherapies, no treatment, and sham or placebo acupuncture to evaluate the efficacy and safety of acupuncture and acupressure for acne vulgaris.

## 2. Methods

Eleven databases were searched from inception to May 2013, with an update in May 2016. Five English (PubMed, Embase, Allied and Complementary Medicine Database (AMED), the Cumulative Index to Nursing and Allied Health Literature (CINAHL), and Cochrane Central Register of Controlled Trials (CENTRAL)) and six Chinese databases (Chinese National Knowledge Infrastructure (CNKI), Chongqing VIP Information Company (CQVIP), Wanfang Data, Chinese Biomedical Literature Database (CBM)) as well as China's Conference Papers Database and China Dissertation database were searched. There were no language restrictions. Search terms included acne vulgaris, papulo-pustular acne, acupuncture, acupressure, moxibustion, auricular acupuncture and auricular acupressure, electro-acupuncture, electro stimulation, and variants. Moxibustion and acupressure were included as they are commonly used techniques to directly stimulate acupuncture points. Moxibustion in particular is commonly combined with acupuncture, and the Chinese term for acupuncture “zhen jiu” literally means acupuncture and moxibustion. Search terms for study design included randomized controlled trials, controlled clinical trials, drug therapy, placebo, and variants.

Titles and abstracts of identified citations were scanned to identify potentially eligible randomized controlled trials (RCTs). Full text was retrieved when eligibility could not be ascertained from the title and abstract. RCTs of acupuncture, acupressure, auricular acupuncture, moxibustion, and electroacupuncture compared to no treatment, sham acupuncture, placebo, or conventional pharmacotherapy for acne vulgaris were included in the review. No age, gender, ethnicity, or language limitations were applied. Trials that included other modalities, as cointervention, such as pharmacotherapy or Chinese medicine techniques other than those specified above were excluded.

The primary outcome was the change in lesion count measured by therapeutic effective rate (TER). Chinese medicine guidelines recommend reporting the TER ≥50% based on lesion count alone or a combination of lesion count and severity [22]. Many of the studies used a TER of ≥30% as an improvement based on Chinese medicine guidelines from 1994 [23]. The criteria for therapeutic effective rate from the 1994 guideline were based on a change in lesion count and associated symptoms. For analysis, we included data for people who achieved 30% or greater on lesion count, irrespective of the minimum threshold used by the study for effectiveness. Secondary outcomes included severity grading, physician's overall grading (physician's assessment or self-reporting), photographic grading, quality of life instruments, and adverse events (AE) reports.

Data extracted included patient demographics, sample size, dropout rate, details of the intervention and comparator, outcome measures, results, and adverse events. Authors were contacted if there was missing data. Verification of data was conducted by an independent researcher (IZ).

Two researchers (KW, IZ) independently assessed methodological quality using Cochrane Collaboration's risk of bias tool [24]. Trials were judged as low, unclear, or high risk of bias for the domains of sequence generation, allocation concealment, blinding of participants, blinding of outcome assessors, incomplete outcome data, selective reporting, and other forms of bias such as conflicts of interest. For acupuncture studies, it is not feasible to blind personnel (practitioner) [25]. Disagreements in judgments were resolved by consulting another reviewer (TZ).

Statistical analyses were performed using Review Manager 5.3.5 [26]. Dichotomous data are presented as risk ratio (RR) and continuous data as mean difference, with 95% confidence intervals (CIs). Data were analyzed for available cases. A random effects model was used. Statistical heterogeneity was considered substantial when the *I*^2^ statistic was greater than 50%. We planned to perform sensitivity analysis with studies assessed as low risk of bias for sequence generation. Subgroup analyses were also conducted on ≥50% and ≥30% TER. Exploration of publication bias was planned if more than ten studies were included in any meta-analysis. Due to the number of included trials and methodological quality, not all planned analyses could be performed.

## 3. Results

### 3.1. Search Results

**Figure 1:**
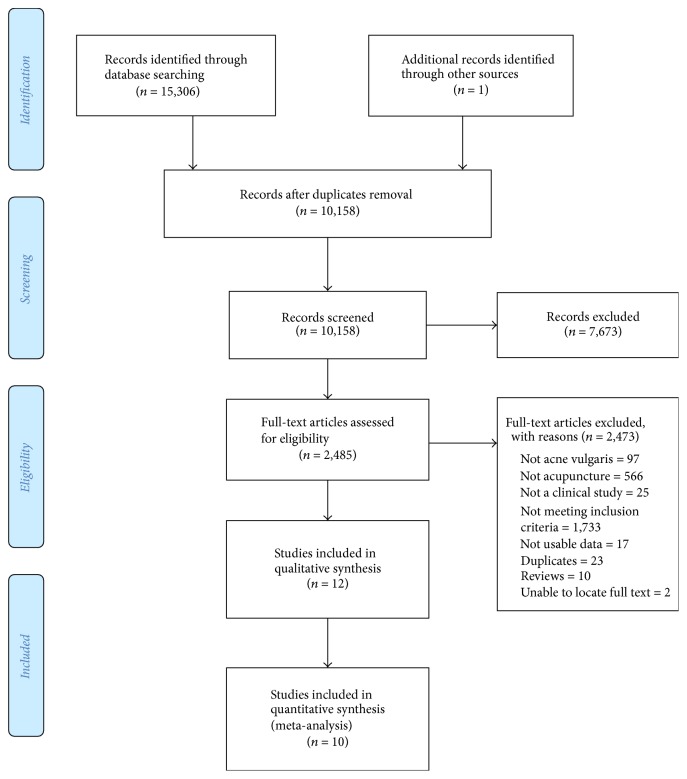
Study selection flow chart.

A total of 15,306 records with one additional record sourced elsewhere were identified from database searches. After removal of duplicates, screening of titles and abstracts excluded 7,673 papers, and 2,485 full texts were reviewed (Figure 1).

### 3.2. Characteristics of Studies

Twelve RCTs involving 1,026 participants met the inclusion criteria [27–38]. Ten RCTs with 975 participants were included in the meta-analysis. The data presented from two trials could not be reanalyzed due to data not being available for individual groups; these were excluded from quantitative analysis [37, 38]. The authors were contacted for additional information; however this was unsuccessful. All trials recruited male and female participants except K. S. Kim and Y.-B. Kim [37] who recruited only male subjects. Participant age ranged from 13 to 37 with a median of mean age of 23.1 years. Details of trial location, treatment times, follow-up periods, and participant stage and duration of condition are presented in Table 1.

The intervention most frequently used was acupuncture (six trials) [27, 28, 31–33, 35] followed by auricular acupressure (two trials) [29, 30]. One trial used electroacupuncture [36] and one trial used acupuncture combined with moxibustion [34]. The comparators are described in Table 2. K. S. Kim and Y.-B. Kim 2012 [37] included three treatment arms, one of acupuncture alone, one of herbal medicine alone, and one where herbal medicine was combined with acupuncture. These three groups were compared with a wait list control. Only the data for the acupuncture arm was included in this analysis. McKee et al. 2004 [38] included two treatment arms, auricular acupuncture and auricular electroacupuncture which were compared to placebo control groups, sham auricular acupuncture, and sham auricular electroacupuncture, respectively.

There was large variation in acupuncture points used (Table 2). Three studies [27, 28, 34] used CV13 Shangwan, CV12 Zhongwan, CV4 Guanyuan, CV6 Qihai, ST24 Huaroumen, ST26 Wailing, Shang Feng Shi Dian (an abdominal point 0.5 cun lateral to ST 24 Huaroumen), and KI13 Qixue. Most of the studies used a standardized set of acupuncture points with one study using a semistandardized approach [30]. Four studies used Ashi points [28, 29, 33, 34] where the location was not specified and two used needles around acne lesions (surrounding acupuncture [29, 33]).

One trial [29] reported on therapeutic effective rate based on lesion count and severity and also reported on serum testosterone and recurrence rate. Four trials [27, 31, 34, 35] reported on therapeutic effective rate according to the 2002 Chinese medicine research guidelines [22]. One trial [33] used the Chinese medicine research guidelines from 1997 [39] and three trials [28, 32, 36] used the 1994 Chinese medicine research guidelines [23]. One trial did not refer to a guideline for judgment of therapeutic effective rate but indicated an improvement of lesion of 95% was a cure and 60% was a significant improvement; these data were included in the meta-analysis [30]. The criteria for determining clinical effect are described in Supplementary Table  1. Only one trial reported measuring quality of life, using Skindex 29 [37].

### 3.3. Risk of Bias

**Figure 2:**
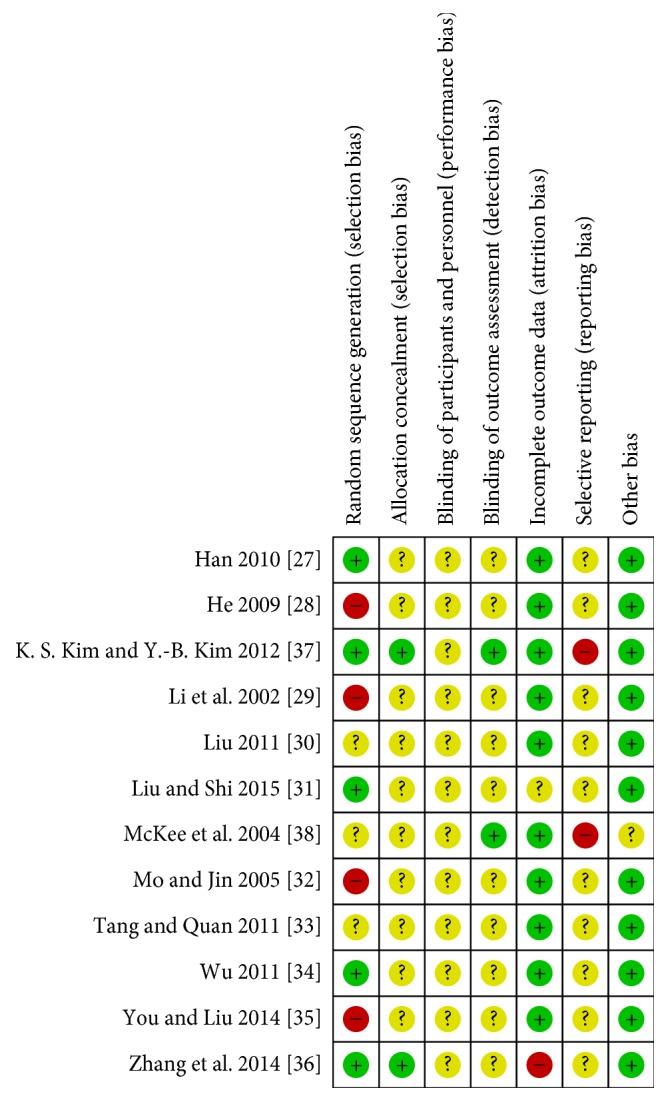
Risk of bias summary.

Methodological quality of the trials was generally low (Figure 2). Four trials [28, 29, 32, 35] were assessed as high risk of bias in the domain of sequence generation as they used sequence of visit for randomization. Five trials [27, 31, 34, 36, 37] were assessed as low risk as random number generators were used. Three trials were assessed as unclear as there was insufficient information [30, 33, 38]. All trials were assessed as unclear risk in blinding of participants. Two trials were assessed as low risk for blinding of outcome assessors [37, 38] and ten were at unclear risk due to insufficient information. One trial was assessed as unclear risk for incomplete data [31] as they did not report dropout data. One trial, Zhang et al. [36], reported on dropout but data was reported only for those who completed the trial and thus was assessed as high risk for incomplete outcome data. Ten trials were assessed as low risk for incomplete data. Two trials were assessed as high risk for selective outcome reporting. McKee et al. [38] stated they would include data on adverse events but no data were presented. K. S. Kim and Y.-B. Kim 2011 indicated in their protocol [40] the use of VAS scale but no data was reported. The remaining ten trials were assessed as unclear as there were no trial protocols published or trial registrations identified [27–36].

### 3.4. Primary Outcome: Therapeutic Effective Rate

**Figure 3:**
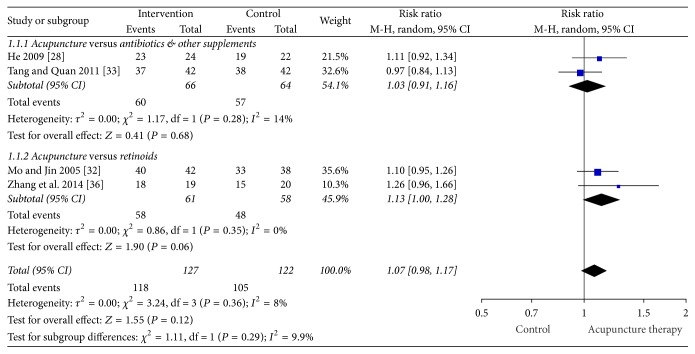
Forest plot of TER ≥30% change in symptoms.

Figure 3 presents the forest plot for TER ≥30% change in symptoms. In the meta-analysis of the trials that defined the TER ≥30% as improvement, the chance of achieving a 30% or greater change in lesion count in the acupuncture group was not different to the combined pharmacotherapy group (retinoids, antibiotics, and other supplements) (four studies, RR: 1.07 [95% CI 0.98, 1.17] and *I*^2^ = 8%) [28, 32, 33, 36] with low heterogeneity. Subgroup analysis of studies where the comparator was antibiotics plus other supplements showed the chance of a 30% or greater change in lesion count was not different between acupuncture and topical/oral antibiotics and supplements group (two studies, RR: 1.03 [95% CI 0.91, 1.16] and *I*^2^ = 14%) [28, 33] with low heterogeneity. In a subgroup analysis of the chance of a change of 30% or greater in lesion count acupuncture was as effective as the retinoids groups (viaminate and tretinoin) (two studies, RR: 1.13 [95% CI 1.00, 1.28], *P* = 0.06, and *I*^2^ = 0%) with no heterogeneity [32, 36].

**Figure 4:**
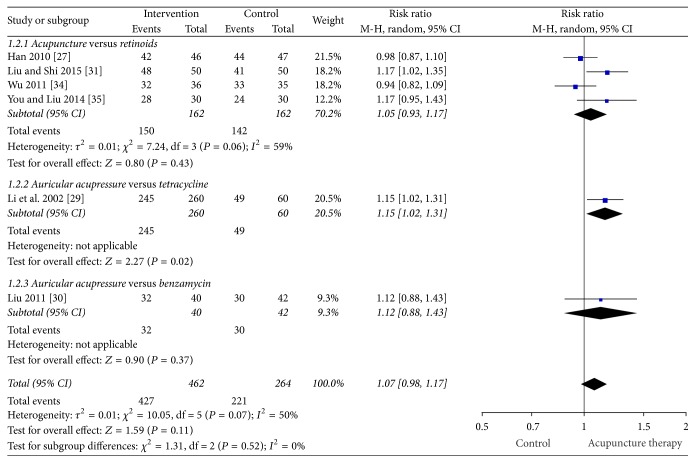
Forest plot of TER ≥50% change in symptoms.

Figure 4 presents the forest plot for TER ≥50% change in symptoms. In the meta-analysis of the data from the trials that used ≥50% TER, the chance of a greater than 50% change in lesion count in the acupuncture group was not statistically different to the pharmacotherapy group (retinoids and antibiotics) (six studies, RR: 1.07 [95% CI 0.98, 1.17] and *I*^2^ = 50%) [27, 29–31, 34, 35]; however there was moderate-to-substantial heterogeneity. In a subgroup analysis, the chance of a 50% or greater change in lesion count in the acupuncture group was not different to the retinoid group (isotretinoin and topical tretinoin) in four studies (four studies, RR: 1.05 [95% CI 0.93, 1.17] and *I*^2^ = 59%) with moderate-to-substantial heterogeneity [27, 31, 34, 35]. Two auricular acupressure trials were not combined due to differences in comparator types (one comparator was an oral pharmaceutical and the other was a topical preparation). Auricular acupressure was more effective compared to oral tetracycline for TER ≥50% (one study, RR: 1.15 [95% CI 1.02, 1.31]) [29]; however there were four times more participants in the intervention group compared to the comparator group with no reasons provided. Another study of auricular acupressure found no benefit compared to topical benzamycin (one study, RR: 1.12 [95% CI 0.88, 11.43]) [30].

### 3.5. Secondary Outcomes

The paper by K. S. Kim and Y.-B. Kim [37] was the only trial to report on quality of life using Skindex 29 score. The data were not presented in a way that permitted reanalysis, so the effects remain unclear. The study authors concluded that the use of acupuncture and Chinese herbal medicine* Keigai-rengyo-to* could be used for inflammatory acne lesions but further research was required.

A total of 127 adverse events were reported in three trials [27, 33, 34]. The other nine did not mention any adverse events. There were more adverse events in the control group (98 in the control group and 29 in the intervention group). Adverse events in the intervention group included painful sensation (11 cases), ecchymosis (nine cases), flushing (five cases), and itchy sensation after needle withdrawal (four cases) which are common adverse events seen after needle penetration and acupressure [41, 42]. In the control group, adverse events that included dry mouth (75 cases), dry skin and desquamation (17 cases), and gastrointestinal discomfort (six cases) are also common adverse events following topical benzoyl peroxide and retinoid treatment [4, 43]. No serious adverse events were reported in the included trials.

## 4. Discussion

This systematic review showed that the chance of ≥30% and ≥50% improvement in acne symptoms with body acupuncture, electroacupuncture, and auricular acupressure was not statistically different from that of pharmaceuticals for acne vulgaris. Interestingly, the magnitude of the treatment effect and the 95% CIs were the same for the primary meta-analyses, regardless of which criteria were used to measure clinical change. There were more adverse events in the pharmacotherapy/control group than in the acupuncture/intervention group. Based on the included studies, acupuncture was well tolerated by participants with acne vulgaris.

While not validated, TER is a common measure of effect in Chinese medicine trials. The TER for acne vulgaris is a subjective outcome that includes a change in lesion count or severity. The Chinese research guidelines for acne from 2002 [22] suggest a ≥50% change in lesion count or severity whereas the 1994 guidelines [23] suggested ≥30% change in lesion count and symptoms. In this review, acupuncture was as effective as antibiotics in trials that used the TER criteria of a ≥30% improvement in symptoms. In the trials that used a ≥50% improvement in symptoms, auricular acupressure was as effective as antibiotics and acupuncture was as effective as topical and oral retinoids. There is currently no consensus on outcome measures for acne though there are efforts underway to standardize them [44]. There was only one trial that reported on quality of life measure Skindex 29 even though there is mounting evidence that sufferers of acne vulgaris may experience considerable psychological and emotional burden [45].

All trials in the quantitative analysis used retinoids or antibiotics as the comparator. Retinoids and antibiotics have demonstrated efficacy for acne [4]; however long term antibiotic use can contribute to antibiotic resistance [46]. Retinoids have severe adverse effects such as teratogenicity and should be used with caution in people of childbearing age [46]. Acupuncture and auricular acupressure were shown in this analysis not to be statistically different to guideline recommended treatments but with fewer side effects and may be an option for those wanting an alternative treatment to pharmaceuticals. Treatment times varied considerably across the trials. Such variations of treatment times could introduce clinical heterogeneity. The typical treatment duration for body acupuncture is 20 to 30 minutes for each treatment and treatment frequency may vary from one to five times per week depending on the local clinical practice environment. Fibromyalgia and tension headache studies have found 20- to 30-minute needle retention, repeated stimulation on acupuncture points (*de-qi* sensation), and daily or twice weekly treatment to have better clinical outcomes compared to less needle retention time and once-per-week treatment [47, 48].

The findings of this review are similar to previous reviews [20, 21]; however previous reviews included trials that compared Chinese medicine interventions against each other such as acupuncture compared to herbal medicines. This review faced the same limitations as others in terms of the methodological quality of included trials. Methodological quality of included studies was low, with four of the twelve studies assessed as high risk of bias and three unclear in the domain of sequence generation. There was also insufficient information on blinding of outcome assessors and participants.

Sample sizes were small, and none of the included studies reported sample size calculations. Not all trials reported on the severity of lesions. There were no follow-up assessments in the included trials. Statistical heterogeneity was also detected in several subgroup analyses which were not able to be explored due to small numbers of studies. Detailed reporting of trial information was lacking; none of the trials addressed all items from Consolidated Standard of Reporting Trials (CONSORT) [49] or Standards for Reporting Interventions in Clinical Trials of Acupuncture (STRICTA) [50] standard reporting conventions. The STRICTA guidelines are important to improve transparency of intervention reporting in acupuncture clinical trials. For studies included in this review, several items were reported well in all trials: the type of acupuncture used, standard acupuncture name and/or locations of acupuncture points, the number and duration of treatment sessions, and the precise descriptions of the controls or comparators (Supplementary Table  2). The trials conducted in China did not provide information about practitioners, the setting and context of treatment, instructions to practitioners, and information and explanations to the patients. This can pose an issue with reproducibility of studies and may be a source of bias. Reporting of such details would enhance accurate analysis and interpretation of data and improve research reliability in acupuncture interventions [51].

## 5. Conclusions

There was no statistical difference in the efficacy of acupuncture compared to pharmacotherapies for acne vulgaris; however acupuncture interventions reported less adverse effects. Poor methodological quality of trial design and lack of consistent reporting of outcome measures from some trials were found in this review; therefore results should be interpreted with caution. Future trials should include rigorous methodological design and reporting should follow standard reporting conventions such as CONSORT and STRICTA. Quality of life measures and further understanding of the mechanisms of acupuncture on acne should also be considered for future studies.

## Figures and Tables

**Table 1:** Characteristics of studies.

First author, publication year	Trial location	Treatment duration, follow-up duration	Stage, severity, and duration of condition	Number of participants randomized/assessed; dropouts or withdrawals	Age (mean (SD) or range); gender (M/F)
Han; 2010 [27]	China, hospital, outpatients	8 w, 1 m	Stage: NS	I: 50/46; 4 C: 50/47; 3	I: 25.83 ± 5.25; 18/28 C: 24.68 ± 4.36; 14/33
			Severity: Pillsbury I, II		
			Duration: I: 2.35 ± 0.86; C: 2.15 ± 0.82		

He; 2009 [28]	China, hospital, outpatients	3 w, NS	Stage: NS	I: 24/24; 0 C: 22/22; 0	I: 25.2; NS C: 23.6; NS
			Severity: slight to severe		
			Duration: I: 20 d to 16 y; C: 1 m to 17 y		

Li; 2002 [29]	NS	6 d, 1 m	Stage: NS	I1: 200/200; 0 I2: 60/60; 0 C: 60/60; 0	I1: 13–37; NS I2: 14–35; NS C: 14–33; NS
			Severity: Samuelson 1–9		
			Duration: I1: 14 d–15 y, I2: 7 d–13 y; C: 4 d–14 y		

Liu; 2011 [30]	NS	2 w, 6 m	Stage: NS	I: 40/40; 0 C: 40/40; 0	I: 14–41; 6/34 C: 13–30; 7/33
			Severity: NS		
			Duration: I: 1 w–14 y; C: 1 w–10 y		

Mo; 2005 [32]	NS	2 w, NS	Stage: NS	I: 42/42; 0 C: 38/38; 0	NS NS
			Severity: NS		
			Duration: NS		

Tang; 2011 [33]	NS	10 d, NS	Stage: NS	I: 42/42; 0 C: 42/42; 0	NS; 11/31 NS; 7/35
			Severity: NS		
			Duration: 1 w–9 y		

Wu; 2011 [34]	NS	8 w, 2 m	Stage: NS	I: 40/36; 4 C: 40/35; 5	I: 24.31 ± 4.08; 13/23 C: 23.91 ± 3.83; 13/22
			Severity: NS		
			Duration: I: 11.92 ± 8.93; C: 12.77 ± 9.58		

Liu; 2015 [31]	NS	8 w, NS	Stage: NS	I: 60/50; 10 C: 58/50; 8	I: 23.2; 27/33 C: 24; 27/31
			Severity: NS		
			Duration: I: 6.5 m; C: 6.1 m		

Zhang; 2014 [36]	NS	4 w, NS	Stage: NS	I: 20/19; 1 C: 20/20; 0	I: 18–23; 2/17 C: 18–24; 5/15
			Severity: NS		
			Duration: I: 6 m–5 y; C: 6 m–4.5 y		

You; 2014 [35]	NS	30 d, NS	Stage: NS	I: 30/30; 0 C: 30/30; 0	I: 25 ± 5; 17/13 C: 25 ± 5; 15/14
			Severity: NS		
			Duration: I: 23.36 m; C: 22.81 m		

McKee; 2004 [38]	USA, outpatient clinic	20 w, NS	Stage: NS	I1: 6/6; 2 I2: 11/11; 6 C1: 6/6; 1 C2: 6/6; 0	I1: F 16 (2.1) M 15 (0.7); NS I2: F 21 (3.4) M 16 (3.6); NS C1: F 19 (4.2) M 17 (1.9); NS C2: F 21 (1.5) M 15 (1.2); NS
			Severity: grade I & II mild-to-moderate nonscarring facial by dermatologist; photographs grading by Cook 1979 and lesion count		
			Duration: NS		

Kim; 2012 [37]	Korea, outpatient clinic	4 w, NS	Stage: NS	I: 11/11, 3 C: 11/11, 2	I: M 21.5 (3.6); NS C: M 23.3 (4.1); NS
			Severity: Korean Acne Grading System grades 2–4 (>10 papules, <20 nodules on face);		
			Duration: >3 months (chronic stage)		

NS: not stated; I: intervention; C: control; F: female; M: male; d: days; w: weeks; m: months; y: years.

**Table 2:** Interventions and comparators.

First author, publication year	Intervention type	Acupuncture points	Intervention treatment frequency	Control details	Control treatment frequency
Han, 2010 [27]	Acupuncture	CV13 Shangwan, CV12 Zhongwan, CV4 Guanyuan, CV6 Qihai; ST24 Huaroumen, ST26 Wailing; Shangfeng Shidian (abdominal point 0.5 cun lateral and superior to ST24); KI13 Qixue, M-CA-23 Sanjiaojiu (Qipang)	30 mins, 3 times per week	Isotretinoin capsules (oral)	10 mg b.i.d. (first month); 10 mg q.d. (second month)

He, 2009 [28]	Acupuncture	CV13 Shangwan, CV12 Zhongwan, CV4 Guanyuan, CV6 Qihai; ST24 Huaroumen, ST26 Wailing; Shangfeng Shidian (abdominal point 0.5 cun lateral and superior to ST24); KI13 Qixue, M-CA-23 Sanjiaojiu (Qipang), M-HN-3 Yintang, SI18 Quanliao, ST4 Dicang, M-HN-9 Taiyang, Ashi points	30 mins: body points; 15–20 mins: head points Ashi points q.d. (first week), every 2 days (2nd and 3rd weeks)	Metronidazole (topical)	b.i.d.

Li, 2002 [29]	I1 AA + SA I2: AA	SA: Ashi points, AA: endocrine, lung, sympathetic, stomach, large intestine, ear Shen Men, internal genitals	SA: 30 min q.d.; AA: 3–5 min, b.i.d.	Tetracycline (oral)	0.5 g, q.i.d.

Liu, 2011 [30]	AA	Lung, endocrine, adrenal gland, ear Shen Men, subcortex, cheek; large intestine (wind and heat in lung meridian); spleen, stomach, large intestine (heat and damp in spleen and stomach); liver, kidney (penetrating and conception meridian disharmony)	AA: 5–10 min, t.i.d.	Benzamycin (oral)	b.i.d.

Mo, 2005 [32]	Acupuncture	Governor meridian	4-5 hrs/treatment, 5 times per week	Viaminate capsules, Vit B6 (oral)	Viaminate 0.25 mg t.i.d.; Vit B6, 2 pills t.i.d.

Tang, 2011 [33]	Acupuncture + SA	Manual: ST36 Zusanli, ST40 Fenglong, ST45 Lidui, LI11 Quchi, LI10 Shoushanli, LI4 Hegu; SA: Ashi points	30 min q.d.	Erythromycin, zinc sulfate (oral); sulphur (topical)	Erythromycin 0.2 g b.i.d.; zinc sulfate 0.2 g b.i.d.; sulphur (topical) q.d.

Wu, 2011 [34]	Acupuncture + moxibustion	Acupuncture: CV11 Shangwan, CV12 Zhongwan, CV4 Guanyuan, CV6 Qihai; ST24 Huaroumen, ST26 Wailing; Shangfeng Shidian (abdominal point 0.5 cun lateral and superior to ST24); KI13 Qixue; Moxa: CV4 Guanyuan, CV6 Qihai	Acupuncture and moxibustion: 3 times per week	Isotretinoin capsules (oral)	10 mg b.i.d.

Liu, 2015 [31]	Acupuncture	GB14 Yangbai, SI18 Quanliao, GV14 Dazhui, LI4 Hegu, LI11 Quchi, ST44 Neiting	q.d. (total of 56 treatments)	Isotretinoin (oral)	10 mg b.i.d.-t.i.d.

Zhang, 2014 [36]	EA	Governor meridians, Jiaji and bladder through the first lateral line, lung, large intestine, stomach	Twice per week (total of 15 treatments)	Tretinoin (topical)	b.i.d.

You, 2014 [35]	Acupuncture	M-HN-3 Yintang, SI18 Quanliao, CV24 Chengjiang, BL13 Feishu, BL19 Ganshu, BL20 Pishu, BL23 Shenshu, CV12 Zhongwan, CV10 Xiawan, CV4 Guanyuan, CV6 Qihai, ST25 Tianshu, GV14 Dazhui, LI11 Quchi, LI4 Hegu, ST36 Zusanli, KI3 Taixi, LU9 Taiyuan	q.d. (total of 15 treatments)	Tretinoin (topical)	q.n.

McKee, 2004 [38]	Auricular acupuncture and EA	I1: Oleson's Shen Men, allergy point, skin disorder point F, point zero, lungs 1 and 2, endocrine point, genital control point, face point bilateral ears; I2: points as I1 plus EA 8–16 sec on 5–80 Hz	20 min weekly	C1: 9 sham points on helix auricular ridge C2: 9 sham points on helix auricular ridge as C1 plus EA 8–16 sec, 10–40 Hz	20 min weekly

Kim, 2012 [37]	Acupuncture	ST2 Sibai, ST6 Jiache, ST36 Zusanli, LI20 Yingxiang, LI11 Quchi, PC6 Neiguan, HT8 Shaofu, SP3 Taibai, SP6 Sanyinjiao, SP10 Xuehai, LR3 Taichong, and/or Ashi points randomly selected at papules and nodules on the face by acupuncture practitioner	Twice weekly for 4 weeks	Waitlist (no treatment)	

I: intervention; C: control; EA: electroacupuncture; q.d.: one time per day; b.i.d.: twice daily; t.i.d.: three times daily; q.i.d.: four times daily; q.n.: once nightly; AA: auricular acupressure; SA: surround needle; sec: seconds; Hz: hertz.

## Supplementary Materials

Supplementary Materials Supplementary Table  1: therapeutic effective rate criteria and secondary outcomes. Supplementary Table  2: assessment of reporting of STRICTA items.
