# Supplementary material for: Acupuncture for Acne Vulgaris: A Systematic Review and Meta-Analysis
Source: Evid Based Complement Alternat Med. 2018 Mar 12;2018:4806734. doi: 10.1155/2018/4806734 (PMC5867647; doi:10.1155/2018/4806734)
Supplement: Supplementary Materials — Supplementary Table 1: therapeutic effective rate criteria and secondary outcomes. Supplementary Table 2: assessment of reporting of STRICTA items. [file 4806734.f1.zip › Supplementary Files Tables 1 and 2 ECAM_ECAM_2156204.docx]

**Supplementary Files**

**Supplementary Table 1: Therapeutic Effective Rate Criteria and Secondary Outcomes**

| First author, publication year | Therapeutic Effective Rate Criteria | Secondary outcomes reported |
| --- | --- | --- |
| Han, 2010 [27] | 2002 guideline  Cure: 90% lesions disappear, all clinical symptoms disappear;  Significant improvement: 60%-89% lesions disappear, clinical symptoms significantly improved;  Improvement: 30-59% lesions disappear, clinical symptoms improved;  No improvement: <30% lesions disappear, or clinical symptoms worsen. | AE:  I: 4 cases of itchy sensation after acupuncture, 7 cases of ecchymosis, 5 cases of painful sensation;  C: 47 cases of dry mouth, 10 cases of dry skin and desquamation. |
| He, 2009 [28] | Cure: All lesions disappear;  Significant improvement: >60% lesions disappear;  Improvement: >30% lesions disappear;  No improvement: <30% lesions disappear, or clinical symptoms worsen. | NS |
| Li, 2002 [29] | Samuelson grading system  If grading >3:  - Significant improvement: grading decrease 3 levels;  - Better improvement: grading decrease 2 levels;  - Improvement: grading decrease 1 level;  - No improvement: grading no change;  - Worsen: if grading increase 1-2 levels.  If grading <3:  - Significant improvement: lesion count decrease 90%;  - Better improvement: lesion count decrease more than 70%;  - Improvement: lesion count decrease more than 50%. | NS |
| Liu, 2011 [30] | Cure: >95% lesions disappear;  Significant improvement: >60% lesions disappear;  Improvement: >20% lesions disappear;  No improvement: <20% lesions disappear. | NS |
| Mo, 2005 [32] | Standard of diagnosis and therapeutic effect of TCM diseases  中医病证诊断疗效标准  Cure: all lesions disappear, all clinical symptoms disappear;  Significant improvement: >70% lesions disappear, clinical symptoms significantly improved;  Improvement: 30-70% lesions disappear, clinical symptoms improved;  No improvement: <30% lesions disappear, or clinical symptoms worsen. | NS |
| Tang, 2011 [33] | Cure: All lesions disappear;  Significant improvement: >80% lesions disappear, new lesions <5, improvement of oily face, slight itchy sensation;  Improvement: >30% lesions disappear, new lesions <10, slight improvement of oily face and itchy sensation;  No improvement: <30% lesions disappear, or clinical symptoms worsen. | AE: C: 6 cases of gastrointestinal discomfort. |
| Wu, 2011 [34] | 2002 guideline  Cure: all lesions disappear, no pigmentation left;  Significant improvement: most of the lesions disappear, 70%-100% decrease of the scores (based on lesion count and severity);  Improvement: part of the lesions disappear, 30-70% decrease of the scores;  No improvement: no improvement of the lesions, and <30% of the scores decrease. | AE:  I: 5 cases of erubescence, 2 cases of ecchymosis, 6 cases of painful sensation;  C: 28 cases of dry mouth, 7 cases of dry skin and slightly desquamation. |
| Liu, 2015 [31] | 2002 Guideline:  Lesion score calculated as change from baseline.  Cure: ≥95%  Most improved: 70-94%  Improved: 50-69%  Not improved: <50%. | NS |
| Zhang, 2014 [36] | 1994 Guideline  Cure: lesions and symptoms disappear;  Improved: ≥30% lesions heal, symptoms improved;  Not improved: <30% lesions heal, symptoms not improved. | AE: I: 3 cases of acne pain. |
| You, 2014 [35] | 2002 Guideline:  Lesion score calculated as change from baseline.  Cure: ≥95%  Most improved: 70-94%  Improved: 50-69%  Not improved: <50%. | Severity grading: GAGS |
| McKee, 2004 [38] | Not applicable | Photographic grading. |
| Kim, 2012 [37] | Not applicable | Skindex-29; photographic grading; Korean Acne Severity Scale. |

Key: I: Intervention; C: Control; AE: adverse events; GAGS: Global Acne Grading System; NS: Not stated

**Supplementary Table 2: Assessment of Reporting of STRICTA items**

| Item | Detail | Han 2010 [27] | He 2009 [28] | Li 2002 [29] | Liu 2011 [30] | Mo 2005 [32] | Tang 2011 [33] | Wu 2011 [34] | Liu 2015 [31] | Zhang 2014 [36] | You 2014 [36] | McKee 2004 [38] | Kim 2012 [37] |
| --- | --- | --- | --- | --- | --- | --- | --- | --- | --- | --- | --- | --- | --- |
| 1. Acupuncture rationale | 1a) Style of acupuncture (e.g. Traditional Chinese Medicine, Japanese, Korean, Western medical, Five Element, ear acupuncture, etc) | Y | Y | Y | Y | Y | Y | Y | Y | Y | Y | Y | Y |
|  | 1b) Reasoning for treatment provided, based on historical context, literature sources, and/or consensus methods, with references where appropriate | Y | N | N | N | N | N | N | N | N | N | Y | Y |
|  | 1c) Extent to which treatment was varied | N | Y | Y | Y | N | N | Y | Y | Y | Y | Y | Y |
| 2. Details of needling | 2a) Number of needle insertions per subject per session (mean and range where relevant) | N | N | N | NA (auricular) | N | N | N | N | NA (plum blossom needle) | N | Y | Y |
|  | 2b) Names (or location if no standard name) of points used (uni/bilateral) | Y | Y | Y | NA | Y | Y | Y | Y | NA | Y | Y | Y |
|  | 2c) Depth of insertion, based on a specified unit of measurement, or on a particular tissue level | N | N | Y | NA | N | N | N | N | NA | N | Auricular | Y |
|  | 2d) Response sought (e.g. *de qi* or muscle twitch response) | N | N | N | NA | N | N | N | Y | NA | Y | Auricular | N |
|  | 2e) Needle stimulation (e.g. manual, electrical) | N | N | N | NA | N | Y | N | Y | NA | Y | Y | Y |
|  | 2f) Needle retention time | Y | Y | Y | NA | Y | Y | N | Y | NA | Y | Y | Y |
|  | 2g) Needle type (diameter, length, and manufacturer or material) | Y | Partial | Partial | NA | Partial | N | Y | Y | NA | Y | Y | Y |
| 3. Treatment regimen | 3a) Number of treatment sessions | Y | Y | Y | Y | Y | Y | Y | Y | Y | Y | Y | Y |
|  | 3b) Frequency and duration of treatment sessions | Y | Y | Y | Y | Y | Y | Y | Y | Y | Y | Y | Y |
| 4. Other components of treatment | 4a) Details of other interventions administered to the acupuncture group (e.g. moxibustion, cupping, herbs, exercises, lifestyle advice) | NA | Y | Y | Y | NA | NA | Y | NA | Y | NA | N | Y |
|  | 4b) Setting and context of treatment, including instructions to practitioners, and information and explanations to patients | NA | N | N | N | NA | NA | N | NA | N | NA | Y | Y |
| 5. Practitioner background | 5) Description of participating acupuncturists (qualification or professional affiliation, years in acupuncture practice, other relevant experience) | N | N | N | N | N | N | N | N | N | N | Y | Y |
| 6. Control or comparator interventions | 6a) Rationale for the control or comparator in the context of the research question, with sources that justify this choice | N | N | N | N | N | N | N | N | N | N | N | N |
|  | 6b) Precise description of the control or comparator. If sham acupuncture or any other type of acupuncture-like control is used, provide details as for Items 1 to 3 above. | Y | Y | Y | Y | Y | Y | Y | Y | Y | Y | Y | Y |

Key: Y: Yes; N: No; NA: Not applicable
