# Supplementary material for: Acupuncture for Acne Vulgaris: A Systematic Review and Meta-Analysis
Source: Evid Based Complement Alternat Med. 2018 Mar 12;2018:4806734. doi: 10.1155/2018/4806734 (PMC5867647; doi:10.1155/2018/4806734)
Supplement: Supplementary Materials — Supplementary Table 1: therapeutic effective rate criteria and secondary outcomes. Supplementary Table 2: assessment of reporting of STRICTA items. [file 4806734.f1.zip › Supplementary Files Tables 1 and 2 ECAM_ECAM_2156205.pdf]

Supplementary Files

Supplementary Table 1: Therapeutic Effective Rate Criteria and Secondary Outcomes

First author, publication year	Therapeutic Effective Rate Criteria	Secondary outcomes reported
**Han, 2010 [27]**	2002 guideline<br>Cure: 90% lesions disappear, all clinical symptoms disappear;<br>Significant improvement: 60%-89% lesions disappear, clinical symptoms significantly improved;<br>Improvement: 30-59% lesions disappear, clinical symptoms improved;<br>No improvement: <30% lesions disappear, or clinical symptoms worsen.	AE:<br>I: 4 cases of itchy sensation after acupuncture, 7 cases of ecchymosis, 5 cases of painful sensation;<br>C: 47 cases of dry mouth, 10 cases of dry skin and desquamation.
**He, 2009 [28]**	Cure: All lesions disappear;<br>Significant improvement: >60% lesions disappear;<br>Improvement: >30% lesions disappear;<br>No improvement: <30% lesions disappear, or clinical symptoms worsen.	NS
**Li, 2002 [29]**	Samuelson grading system<br>If grading >3:<br>- Significant improvement: grading decrease 3 levels;<br>- Better improvement: grading decrease 2 levels;<br>- Improvement: grading decrease 1 level;<br>- No improvement: grading no change;<br>- Worsen: if grading increase 1-2 levels.<br><br>If grading <3:<br>- Significant improvement: lesion count decrease 90%;<br>- Better improvement: lesion count decrease more than 70%;<br>- Improvement: lesion count decrease more than 50%.	NS
**Liu, 2011 [30]**	Cure: >95% lesions disappear;<br>Significant improvement: >60% lesions disappear;<br>Improvement: >20% lesions disappear;<br>No improvement: <20% lesions disappear.	NS
**Mo, 2005 [32]**	Standard of diagnosis and therapeutic effect of TCM diseases<br>中医病证诊断疗效标准<br>Cure: all lesions disappear, all clinical symptoms disappear;	NS

First author, publication year	Therapeutic Effective Rate Criteria	Secondary outcomes reported
	Significant improvement: >70% lesions disappear, clinical symptoms significantly improved;<br>Improvement: 30-70% lesions disappear, clinical symptoms improved;<br>No improvement: <30% lesions disappear, or clinical symptoms worsen.	
**Tang, 2011 [33]**	Cure: All lesions disappear;<br>Significant improvement: >80% lesions disappear, new lesions <5, improvement of oily face, slight itchy sensation;<br>Improvement: >30% lesions disappear, new lesions <10, slight improvement of oily face and itchy sensation;<br>No improvement: <30% lesions disappear, or clinical symptoms worsen.	AE: C: 6 cases of gastrointestinal discomfort.
**Wu, 2011 [34]**	2002 guideline<br>Cure: all lesions disappear, no pigmentation left;<br>Significant improvement: most of the lesions disappear, 70%-100% decrease of the scores (based on lesion count and severity);<br>Improvement: part of the lesions disappear, 30-70% decrease of the scores;<br>No improvement: no improvement of the lesions, and <30% of the scores decrease.	AE:<br>I: 5 cases of erubescence, 2 cases of ecchymosis, 6 cases of painful sensation;<br>C: 28 cases of dry mouth, 7 cases of dry skin and slightly desquamation.
**Liu, 2015 [31]**	2002 Guideline:<br>Lesion score calculated as change from baseline.<br>Cure: ≥95%<br>Most improved: 70-94%<br>Improved: 50-69%<br>Not improved: <50%.	NS
**Zhang, 2014 [36]**	1994 Guideline<br>Cure: lesions and symptoms disappear;<br>Improved: ≥30% lesions heal, symptoms improved;<br>Not improved: <30% lesions heal, symptoms not improved.	AE: I: 3 cases of acne pain.
**You, 2014 [35]**	2002 Guideline:<br>Lesion score calculated as change from baseline.<br>Cure: ≥95%<br>Most improved: 70-94%<br>Improved: 50-69%<br>Not improved: <50%.	Severity grading: GAGS

First author, publication year	Therapeutic Effective Rate Criteria	Secondary outcomes reported
**McKee, 2004 [38]**	Not applicable	Photographic grading.
**Kim, 2012 [37]**	Not applicable	Skindex-29; photographic grading; Korean Acne Severity Scale.

Key: I: Intervention; C: Control; AE: adverse events; GAGS: Global Acne Grading System; NS: Not stated

Supplementary Table 2: Assessment of Reporting of STRICTA items

Item	Detail	Han 2010 [27]	He 2009 [28]	Li 2002 [29]	Liu 2011 [30]	Mo 2005 [32]	Tang 2011 [33]	Wu 2011 [34]	Liu 2015 [31]	Zhang 2014 [36]	You 2014 [36]	McKee 2004 [38]	Kim 2012 [37]
**1. Acupuncture rationale**	1a) Style of acupuncture (e.g. Traditional Chinese Medicine, Japanese, Korean, Western medical, Five Element, ear acupuncture, etc)	Y	Y	Y	Y	Y	Y	Y	Y	Y	Y	Y	Y
	1b) Reasoning for treatment provided, based on historical context, literature sources, and/or consensus methods, with references where appropriate	Y	N	N	N	N	N	N	N	N	N	Y	Y
	1c) Extent to which treatment was varied	N	Y	Y	Y	N	N	Y	Y	Y	Y	Y	Y
**2. Details of needling**	2a) Number of needle insertions per subject per session (mean and range where relevant)	N	N	N	NA (auricular)	N	N	N	N	NA (plum blossom needle)	N	Y	Y
	2b) Names (or location if no standard name) of points used (uni/bilateral)	Y	Y	Y	NA	Y	Y	Y	Y	NA	Y	Y	Y
	2c) Depth of insertion, based on a specified unit of measurement, or on a particular tissue level	N	N	Y	NA	N	N	N	N	NA	N	Auricular	Y
	2d) Response sought (e.g. *de qi* or muscle twitch response)	N	N	N	NA	N	N	N	Y	NA	Y	Auricular	N

Item	Detail	Han 2010 [27]	He 2009 [28]	Li 2002 [29]	Liu 2011 [30]	Mo 2005 [32]	Tang 2011 [33]	Wu 2011 [34]	Liu 2015 [31]	Zhang 2014 [36]	You 2014 [36]	McKee 2004 [38]	Kim 2012 [37]
	2e) Needle stimulation (e.g. manual, electrical)	N	N	N	NA	N	Y	N	Y	NA	Y	Y	Y
	2f) Needle retention time	Y	Y	Y	NA	Y	Y	N	Y	NA	Y	Y	Y
	2g) Needle type (diameter, length, and manufacturer or material)	Y	Partial	Partial	NA	Partial	N	Y	Y	NA	Y	Y	Y
**3. Treatment regimen**	3a) Number of treatment sessions	Y	Y	Y	Y	Y	Y	Y	Y	Y	Y	Y	Y
	3b) Frequency and duration of treatment sessions	Y	Y	Y	Y	Y	Y	Y	Y	Y	Y	Y	Y
**4. Other components of treatment**	4a) Details of other interventions administered to the acupuncture group (e.g. moxibustion, cupping, herbs, exercises, lifestyle advice)	NA	Y	Y	Y	NA	NA	Y	NA	Y	NA	N	Y
	4b) Setting and context of treatment, including instructions to practitioners, and information and explanations to patients	NA	N	N	N	NA	NA	N	NA	N	NA	Y	Y
**5. Practitioner background**	5) Description of participating acupuncturists (qualification or professional affiliation, years in acupuncture practice, other relevant experience)	N	N	N	N	N	N	N	N	N	N	Y	Y

Item	Detail	Han 2010 [27]	He 2009 [28]	Li 2002 [29]	Liu 2011 [30]	Mo 2005 [32]	Tang 2011 [33]	Wu 2011 [34]	Liu 2015 [31]	Zhang 2014 [36]	You 2014 [36]	McKee 2004 [38]	Kim 2012 [37]
**6. Control or comparator interventions**	6a) Rationale for the control or comparator in the context of the research question, with sources that justify this choice	N	N	N	N	N	N	N	N	N	N	N	N
	6b) Precise description of the control or comparator. If sham acupuncture or any other type of acupuncture-like control is used, provide details as for Items 1 to 3 above.	Y	Y	Y	Y	Y	Y	Y	Y	Y	Y	Y	Y

Key: Y: Yes; N: No; NA: Not applicable
